# Braden scale for assessing pneumonia after acute ischaemic stroke

**DOI:** 10.1186/s12877-019-1269-x

**Published:** 2019-10-07

**Authors:** Yunlong Ding, Yazhou Yan, Jiali Niu, Yanrong Zhang, Zhiqun Gu, Ping Tang, Yan Liu

**Affiliations:** 1 Department of Neurology, Jingjiang People’s Hospital, the Seventh Affiliated Hospital of Yangzhou University, No. 28, Zhongzhou Road, Jingjiang, CN 214500 Jiangsu China; 2 Department of Neurosurgery, Changhai Hospital affiliated to the Second Military Medical University, Shanghai, China; 3 Department of Clinical Pharmacy, Jingjiang People’s Hospital, the Seventh Affiliated Hospital of Yangzhou University, Jingjiang, Jiangsu China

**Keywords:** The Braden scale, Acute ischaemic stroke, Pneumonia

## Abstract

**Background:**

The prevention of pneumonia is critical for patients with acute ischaemic stroke (AIS). The six subscales in the Braden Scale seem to be related to the occurrence of pneumonia. We aimed to evaluate the feasibility of using the Braden Scale to predict the occurrence of pneumonia after AIS.

**Methods:**

We studied a series of consecutive patients with AIS who were admitted to the hospital. The cohort was subdivided into pneumonia and no pneumonia groups. The scores on the Braden Scale, demographic characteristics and clinical characteristics were obtained and analysed by statistical comparisons between the two groups. We investigated the predictive validity of the Braden Scale by receiver operating characteristic (ROC) curve analysis.

**Results:**

A total of 414 patients with AIS were included in this study. Of those 414 patients, 57 (13.8%) patients fulfilled the criteria for post-stroke pneumonia. There were significant differences in age and histories of chronic obstructive pulmonary disease (COPD), dysphagia and Glasgow Coma Scale (GCS) score between the two groups, and the National Institutes of Health Stroke Scale (NIHSS) score in the pneumonia group was significantly higher than that in the no pneumonia group (*P* < 0.01). The mean score on the Braden Scale in the pneumonia group was significantly lower than that in the no pneumonia group (*P* < 0.01). The six subscale scores on the Braden Scale were all significantly different between the two groups. The area under the curve (AUC) for the Braden Scale for the prediction of pneumonia after AIS was 0.883 (95% *CI* = 0.828–0.937). With 18 points as the cutoff point, the sensitivity was 83.2%, and the specificity was 84.2%.

**Conclusion:**

The Braden Scale with 18 points as the cutoff point is likely a valid clinical grading scale for predicting pneumonia after AIS at presentation. Further studies on the association of the Braden Scale score with stroke outcomes are needed.

## Background

Currently, ischaemic stroke is one of the most important causes of death and disability in China, which results in substantial social and economic burdens [1]. Pneumonia is a common medical complication after acute ischaemic stroke (AIS) [2, 3], resulting in a longer length of hospital stay and higher risks of mortality and morbidity [4]. Effective prevention is more critical than the treatment of pneumonia. Factors that have been associated with pneumonia after AIS include older age, dysarthria/aphasia, cognitive impairment, stroke severity, long-term bedridden status, dysphagia, and decreased body resistance [5, 6]. We hope to find an effective scale to predict the risk of pneumonia in patients with AIS according to these risk factors.

The Braden Score is an important assessment method for judging the risk of pressure ulcers, and it involves six different risk factors: sensory perception, skin moisture, activity, mobility, nutrition, and friction and shear [7]. These indexes in the Braden Scale seem to be related to the occurrence of pneumonia. In this paper, we retrospectively analysed the correlation between the Braden Scale score and pneumonia after AIS in the stroke centre of our hospital, to evaluate the feasibility of using the Braden Scale to predict the occurrence of pneumonia after AIS.

## Methods

### Study participants

This retrospective study included AIS patients who were admitted to the stroke centre of our hospital between December 2015 and December 2018. The inclusion criteria were as follows: 1) aged ≥18 years; 2) hospitalized with the primary diagnosis of AIS according to the World Health Organization criteria [8]; and 3) AIS confirmed by brain CT or MRI. The exclusion criteria were as follows: 1) transient ischaemic attack or subarachnoid haemorrhage and 2) pneumonia that occurred before admission. Pneumonia after AIS was diagnosed according to the Centers for Disease Control and Prevention criteria [9] for hospital-acquired pneumonia, on the basis of clinical and laboratory indexes of respiratory tract infection (fever, productive cough with purulent sputum, auscultatory respiratory crackles, bronchial breathing, or positive sputum culture) and supported by abnormal chest radiographic findings.

### Data collection

Demographic and clinical characteristics were obtained at admission including demographic data (age and sex), associated risk factors (hypertension, hyperlipidaemia, diabetes, past stroke or transient ischaemic attack history, history of smoking and drinking, history of chronic obstructive pulmonary disease (COPD), dysphagia and the Glasgow Coma Scale (GCS) score), physical examination results (systolic blood pressure and diastolic blood pressure), laboratory examination results (total cholesterol, triglyceride, low-density lipoprotein cholesterol, high-density lipoprotein cholesterol, fasting blood glucose, glycosylated haemoglobin and serum creatinine levels), the aetiological classification of the ischaemic stroke (large atherosclerotic stroke, arteriolar occlusive stroke, cardiogenic cerebral embolism, other stroke with definite aetiology and stroke of unknown aetiology) and the National Institutes of Health Stroke Scale (NIHSS) score.

The Braden Scale is measured 24 h after admission by nurses and is composed of six subscales: sensory perception, skin moisture, activity, mobility, nutrition, and friction and shear. The minimum score for each item is 1 (worst), and the maximum score is 4 (best), except for the scores for friction and shear, which range from 1 to 3. The summed scores range from 6 to 23, with lower scores associated with a higher risk [10].

### Statistical analysis

Statistical comparisons were made for pneumonia versus no pneumonia after AIS. For normally distributed continuous variables (described as the mean ± SD), analysis was performed using unpaired Student’s *t* tests. For nonnormally distributed continuous variables, analysis was performed using the Mann-Whitney *U* test. Categorical variables were analysed by the chi-square test or *Fisher’s* exact test. Statistical analysis was performed using SPSS version 21.0 (SPSS Inc., Chicago, IL, USA). A *P*-value < 0.05 was considered statistically significant. Then, we investigated the predictive validity of the Braden Scale for pneumonia after AIS by receiver operating characteristic (ROC) curve analysis. An area under the curve (AUC) of 0.97–1.00 indicates excellent accuracy; 0.93 to 0.96 indicates very good accuracy; and 0.75 to 0.92 indicates good accuracy. However, an AUC < 0.75 indicates obvious deficiencies, and an AUC of 0.5 indicates that the test has no predictive ability [11].

## Results

### Subject characteristics

In total, 525 patients with AIS were admitted to the stroke centre of our hospital between December 2015 and December 2018. Among them, 56 patients were discharged from the hospital within 2 days, and 55 patients had incomplete or missing data. Finally, 414 patients with AIS were included in this study. A total of 57 of the 414 (13.8%) patients fulfilled the criteria for hospital-acquired pneumonia, and 357 (86.2%) had no pneumonia. The study population had a mean age of 71.5 years, ranging from 50 to 89 years. Almost 63.8% of the patients (264) were men, and 36.2% of the patients (150) were women.

### Correlations of demographic and clinical characteristics between two groups

The demographic data (sex), associated vascular risk factors (hypertension, hyperlipidaemia, diabetes, past stroke or transient ischaemic attack history, history of smoking and drinking), physical examination (systolic blood pressure, diastolic blood pressure), laboratory examination (total cholesterol, triglyceride, low-density lipoprotein cholesterol, high-density lipoprotein cholesterol, fasting blood glucose, glycosylated haemoglobin, and serum creatinine levels) had no significant differences between the pneumonia and no pneumonia groups. There were significant differences in age, history of COPD, dysphagia and GCS score between the two groups. A significant difference also emerged between the two groups in the NIHSS score, which was significantly higher in the pneumonia group than in the no pneumonia group (13.6 ± 5.0 vs 9.2 ± 3.6, *P* < 0.01). (Table 1).

**Table 1:** Demographic and clinical characteristics of the two groups

Items	No pneumonia (*n* = 357)	Pneumonia (*n* = 57)	*P* value
Age/year	71.0 ± 8.9	74.7 ± 7.5	0.001
Male (case, %)	225 (63.0)	39 (68.4)	0.462
Smoking status (case, %)	140 (39.2)	28 (49.1)	0.191
Drinking status (case, %)	147 (27.4)	22 (38.6)	0.773
Hypertension (case, %)	195 (54.6)	34 (59.6)	0.642
Hyperlipidemia (case, %)	101 (28.3)	12 (21.1)	0.336
Diabetes (case, %)	96 (26.9)	18 (31.6)	0.523
Stroke/TIA (case, %)	36 (10.1)	5 (8.8)	1.000
COPD (case, %)	6 (1.7)	4 (7.0)	0.036
Dysphagia (case, %)	54 (15.1)	18 (31.6)	0.004
Admission GCS score	14.0 ± 2.2	10.9 ± 4.3	0.000
Fasting blood glucose (mmol/L)	5.9 ± 1.6	5.8 ± 0.76	0.255
Glycosylated hemoglobin (%)	5.8 ± 0.7	5.8 ± 0.7	0.822
Serum creatinine (umol/L)	80.4 ± 20.6	82.0 ± 25.1	0.595
Systolic blood pressure (mmHg)	145.5 ± 17.7	143.1 ± 17.4	0.342
Diastolic blood pressure (mmHg)	87.3 ± 10.9	89.4 ± 10.0	0.189
Total cholesterol (mmol/L)	5.0 ± 1.2	5.2 ± 1.6	0.329
Triglyceride (mmol/L)	1.5 ± 0.8	1.4 ± 0.5	0.594
Low density lipoprotein cholesterol (mmol/L)	3.1 ± 0.8	3.1 ± 0.8	0.955
High density lipoprotein cholesterol (mmol/L)	1.2 ± 0.5	1.2 ± 0.4	0.571
Stroke classification			
Large atherosclerotic stroke(case, %)	140 (39.2)	26 (45.6)	0.384
Arteriolar occlusive stroke(case, %)	90 (25.2)	19 (33.3)	0.199
Cardiogenic cerebral embolism(case, %)	49 (13.7)	3 (5.3)	0.085
Other stroke with definite etiology(case, %)	34 (10.0)	3 (5.3)	0.452
Stroke of unknown etiology(case, %)	44 (12.3)	6 (10.5)	0.829
Admission NIHSS score	9.2 ± 3.6	13.6 ± 5.0	0.000
Braden scale at 24 h	19.6 ± 2.3	15.3 ± 2.5	0.000

The NIHSS score in the pneumonia group was significantly higher than that in the no pneumonia group. The mean score on the Braden Scale in the pneumonia group was significantly lower than that in the no pneumonia group

*TIA* transient ischaemic attack, *COPD* chronic obstructive pulmonary disease, *GCS* Glasgow Coma Scale, *NIHSS* National Institutes of Health Stroke Scale

The mean score on the Braden Scale in the pneumonia group was 15.263 ± 2.579, which was significantly lower than that in the no pneumonia group (19.546 ± 2.265, *P* < 0.05). (Table 1) The six subscale scores on the Braden Scale all had significant differences between the two groups. (Table 2).

**Table 2:** The Braden Scale scores in the two groups (mean ± SD)

Braden scale	No pneumonia (*n* = 357)	Pneumonia (*n* = 57)	*P* value
Sensory perception	3.7 ± 0.5	2.8 ± 0.7	0.000
Skin moisture	4.0 ± 0.2	3.7 ± 0.6	0.000
Activity	3.0 ± 1.0	1.6 ± 1.0	0.000
Mobility	3.5 ± 0.6	2.5 ± 0.6	0.000
Nutrition	3.0 ± 0.3	2.8 ± 0.4	0.001
Friction and shear	2.4 ± 0.6	1.9 ± 0.5	0.000
Sum score	19.6 ± 2.3	15.3 ± 2.5	0.000

The scores on the six subscales of the Braden Scale were all significantly different between the two groups

### The validity of the association between the Braden scale/NIHSS score and pneumonia after acute ischaemic stroke

**Fig. 1:**
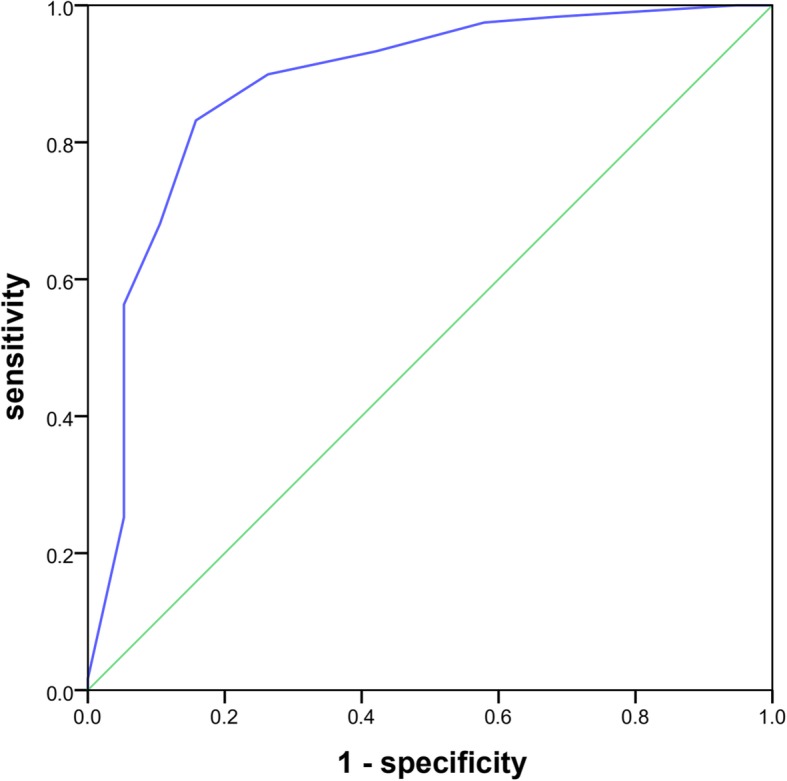
ROC curve for the Braden Scale. The AUC for the Braden Scale for the prediction of pneumonia after acute ischaemic stroke was 0.883 (95% *CI* = 0.828–0.937). With 18 points as the cutoff point, the sensitivity was 83.2%, and the specificity was 84.2%

The AUC for the Braden Scale for the prediction of pneumonia after acute ischaemic was 0.883 (95% CI = 0.828–0.937). Additionally, with 18 points as the cutoff point, the sensitivity was 83.2%, and the specificity was 84.2%. It was suggested that the incidence of pneumonia in patients with AIS can be predicted by a cutoff value of 18 points on the Braden Scale, with a sensitivity of 83.2% and a specificity of 84.2%. (Fig. 1).

**Fig. 2:**
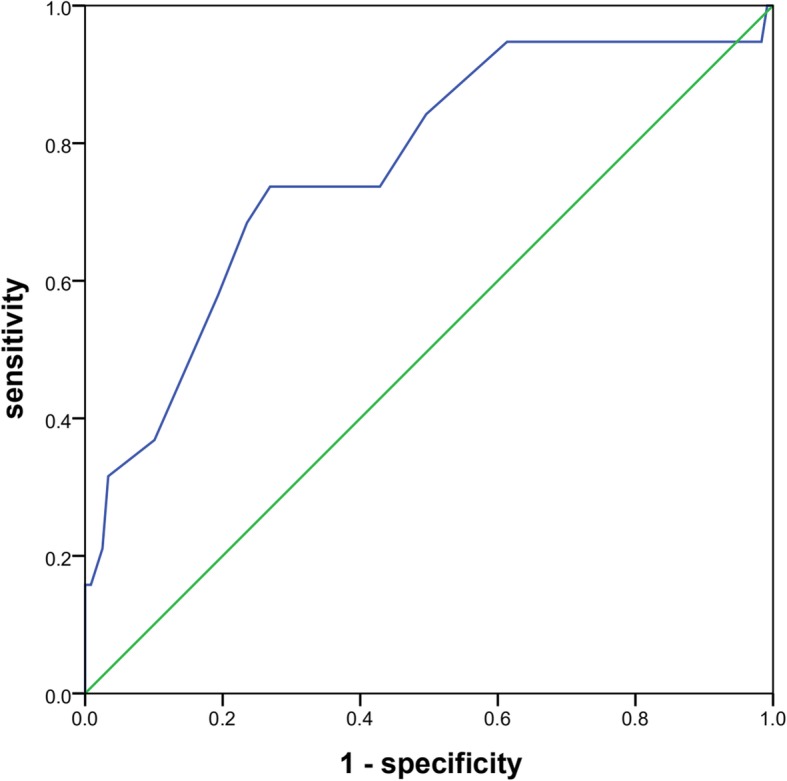
ROC curve for the NIHSS score. The AUC for the NIHSS score for the prediction of pneumonia after acute ischaemic stroke was 0.767 (95% CI = 0.697–0.837). With 12 points as the cutoff point, the sensitivity was 73.7%, and the specificity was 73.1%

The AUC for the NIHSS score for the prediction of pneumonia after AIS was 0.767 (95% CI = 0.697–0.837). With 12 points as the cutoff point, the sensitivity was 73.7%, and the specificity was 73.1%. (Fig. 2).

## Discussion

The primary objective of the present study was to find an effective and simple scale to identify patients at high risk of pneumonia after AIS. This was the first study to evaluate the feasibility of using the Braden Scale to predict the occurrence of pneumonia after AIS. Stroke is one of the leading causes of death at the national level in China [12]. Ageing is an important risk factor for stroke [13], and as life expectancy increases, the incidence of stroke also rises. Therefore, exploring the prevention and treatment of stroke and stroke complications is important for reducing the mortality rate of stroke patients.

In this study, pneumonia was found in 13.8% of patients presenting with an AIS, which was similar to the incidence in prior studies, which ranged from 5 to 26% [14–17]. Post-stoke pneumonia is associated with reduced early and long-term survival, longer hospitalization times, and higher degrees of disability at discharge [4]. Therefore, it is very important to prevent post-stoke pneumonia. However, a systematic review on the efficacy of early antibiotic prophylaxis after stroke failed to show a benefit in patients’ outcomes [18]. This might be due to the inclusion of patients with a low risk of developing post-stoke pneumonia in these studies. It is critical to find an effective scale to predict the occurrence of pneumonia in patients after AIS and to intervene in high-risk patients to prevent pneumonia and improve the outcome. The Braden Scale is composed of six subscales, namely, sensory perception, skin moisture, activity, mobility, nutrition, friction and shear, which seem to be related to the occurrence of pneumonia. One study found that the Braden Scale score can predict the prognosis of elderly people with mobility impairment [19], and our study found that the mean score on the Braden Scale in the pneumonia group was significantly lower than that in the no pneumonia group. Furthermore the scores on the six subscales of the Braden Scale were significantly different between the two groups. The AUC for the Braden Scale for the prediction of post-stoke pneumonia was 0.883, which was identified as good accuracy, as shown above. With 18 points as the cutoff point, the sensitivity and specificity were high. Given that patients with lower Braden scores are at high risk for SAP, they should be screened in a timely fashion and receive early interventions to achieve the goal of reducing SAP. In addition, the use of the Braden Scale score allows medical staff to more accurately identify patients at high risk for developing SAP, increasing clinical care efficiency.

We also found that the NIHSS score in the pneumonia group was significantly higher than that in the no pneumonia group. Studies have shown that the NIHSS score is an independent risk factor for pneumonia after acute stroke [16, 20–22]. The occurrence of pneumonia in patients with a higher NIHSS score may be due to decreased consciousness or to position-induced gastroesophageal reflux. This result also suggested that the pneumonia group had a greater neurological deficit. Previous studies confirmed that patients with cardiogenic embolism tended to have more neurological deficits [23], and our study supported the conclusion that patients with cardiogenic embolism are more likely to develop pneumonia. However, the Braden Scale was better able to quantify the risk factors and evaluate the incidence of post-stoke pneumonia.

Several post-stoke pneumonia prediction models have been developed (see Table 3 for an overview of these models); however, these models have not been widely used in clinical practice. It is not our intention to show the superiority of the Braden Scale for the prediction of the occurrence of post-stoke pneumonia compared to the earlier scores; however, we want to point out the differences. Three of these prediction models were derived from and externally validated in large stroke registries: Hoffmann et al. [24], Ji et al. [25] and Smith et al. [26]. The other available models for predicting post-stroke pneumonia showed worse performance or over-fitting of the model because of their smaller sample sizes, and they often include too many predictors based on the event per variable rule [6, 19, 27–30].

**Table 3:** Models to predict post-stoke pneumonia

Author, year	Study design	No. of patients	Predictors	C-statistic
Kwon et al., 2006 [20]	Retrospective cohort	286	Age, sex, NIHSS, dysphagia, mechanical ventilation	NR
Sellars et al., 2007 [6]	Retrospective cohort	412	Age, dysarthria, abbreviated mental test score, modified Rankin Scale score, and water swallowing test	0.90
Chumbler et al., 2010 [27]	Retrospective cohort	925	Age, stroke severity, dysphagia, history of pneumonia, patient being ‘found down’ at symptom onset	0.78
Hoffmann et al., 2012 [24]	Registry	15,336	Age, sex, stroke severity, dysphagia, atrial fibrillation	0.84
Ji et al., 2013 [25]	Registry	8820	Age, history of atrial fibrillation, congestive heart failure, COPD, current smoking, restroke dependence, dysphagia, NIHSS, GCS, stroke subtype, blood glucose	0.79
Harms et al., 2013 [28]	RCT	114	Age, GCS, systolic arterial blood pressure, WBC count	0.85
Smith et al., 2015 [26]	Registry	11,551	Age, sex, NIHSS, prestrike independence	0.79
Kumar et al., 2017 [29]	Retrospective cohort	1644	Age, congestive heart failure, dysarthria, dysphagia	0.82
Westendorp et al., 2018 [30]	RCT	2538	Age, sex, pre-stroke disability, medical history of COPD, stroke severity, dysphagia, intracerebral haemorrhage	0.82
Ding et al., 2019	Retrospective cohort	414	Sensory perception, skin moisture, activity, mobility, nutrition, and friction and shear	0.88

*NIHSS* National Institutes of Health Stroke Scale, *NR* not reported, *COPD* chronic obstructive pulmonary disease, *GCS* Glasgow Coma Scale, *RCT* randomized controlled trial

Our study had some limitations. First, as a retrospective study, we cannot rule out the possibility that some other confounding factors may have impacted the development of post-stroke pneumonia, such as dementia [31, 32], the use of angiotensin-converting enzyme inhibitors or angiotensin receptor blockers [33]. Second, the time course for post-stroke pneumonia was unclear. We cannot conclude the causal relationship between longer hospital stay and pneumonia. Third, the study included only hospitalized patients with AIS, and those patients who were treated in outpatient clinics, were treated in the emergency department, or died shortly after admission were not included. Fourth, our study was from a single centre with a limited number of patients. Finally, the use of the Braden Scale for the prediction of the occurrence of post-stoke pneumonia needs to be further validated in additional populations.

## Conclusion

In summary, the Braden Scale with 18 points as the cutoff point is a valid clinical grading scale for predicting pneumonia after AIS at presentation. Further studies on the association of the Braden Scale score with stroke outcomes are needed.

## Data Availability

The datasets analysed during the current study are available from the corresponding author on reasonable request.

## Abbreviations

AIS: Acute Ischaemic Stroke
AUC: Area Under the Curve
COPD: Chronic Obstructive Pulmonary Disease
GCS: Glasgow Coma Scale
NIHSS: National Institutes of Health Stroke Scale
ROC: Receiver Operating Curve
TIA: Transient Ischaemic Attack
